# Navigating the Perils of Anesthesia: Managing Mediastinal Masses for Tru-Cut Biopsy

**DOI:** 10.7759/cureus.65426

**Published:** 2024-07-26

**Authors:** Sonal Khatavkar, Veda Sumi Durgumpudi

**Affiliations:** 1 Anesthesiology, Dr. D. Y. Patil Medical College, Hospital & Research Centre, Dr. D. Y. Patil Vidyapeeth, Pune, IND

**Keywords:** tru-cut biopsy, mediastinal carcinoma, mediastinal mass diagnosis, anaesthesiology pediatric anaesthesia, mediastinal mass

## Abstract

Managing mediastinal masses during anesthesia presents formidable challenges, particularly in pediatric patients undergoing procedures such as tru-cut biopsy. These masses, both benign and malignant, can compress vital structures, leading to life-threatening complications. This article explores the complexities of managing anesthesia in patients with mediastinal masses, emphasizing the importance of meticulous preoperative assessment, understanding the relationship between the mass and surrounding anatomy, and employing lifesaving techniques such as inhalation induction and awake intubation. In the first case, a seven-year-old boy with a large heterogeneous mediastinal mass causing left lung collapse and compression of major vessels underwent a tru-cut biopsy under spontaneous general anesthesia. The procedure was uneventful, and the mass was diagnosed as neuroblastoma. In the second case, a 13-year-old boy with a mediastinal mass causing compression of the trachea and major vessels presented with respiratory distress and was managed with a tru-cut biopsy under local anesthesia with ultrasound guidance. The mass was diagnosed as acute T-cell lymphoblastic lymphoma. In the third case, a 14-year-old girl with a large mediastinal mass causing compression of the pulmonary trunk and major vessels experienced airway compromise during the biopsy, necessitating emergency intubation and repositioning. The mass was diagnosed as Hodgkin lymphoma. Mediastinal masses can cause significant compression of the trachea, bronchi, and major vessels, leading to a range of clinical symptoms. Effective management requires thorough preoperative evaluation, planning for potential airway emergencies, and collaboration with surgical teams. Case reviews highlight the variability of airway dynamics and the necessity of positive pressure ventilation and vigilant postoperative monitoring. Comprehensive pre-procedural assessment, preparedness for airway emergencies, and skilled anesthesia teams are crucial for managing pediatric patients with mediastinal masses. These cases underscore the complexities and emphasize the importance of careful planning and proactive measures to ensure successful outcomes and minimize risks during anesthesia induction and diagnostic procedures.

## Introduction

In the realm of anesthesia, few challenges are as formidable as those presented by mediastinal masses. This heterogeneous group of tumors, comprising both benign and malignant entities, poses a grave threat to patients undergoing surgery, particularly when general anesthesia is involved. The stakes are elevated, with the potential for life-threatening complications stemming from compression of vital airways and vascular structures. Understanding the intricate relationship between these masses and surrounding anatomy, coupled with meticulous preoperative assessment and collaboration with surgical teams, is paramount for ensuring successful outcomes. Traditional approaches, such as inhalation induction and awake intubation, are considered lifesaving measures, yet the evidence supporting these techniques remains largely anecdotal, drawn from case reports and limited case series. Despite the inherent limitations of such evidence, the urgency of the matter demands attention, prompting the continued publication and practice of recommendations for safe anesthesia induction. In this article, we delve into the complexities of three mediastinal mass cases, exploring their pathophysiology, clinical manifestations, and strategies for mitigating perioperative risks.

## Case presentation

Case 1

**Figure 1:**
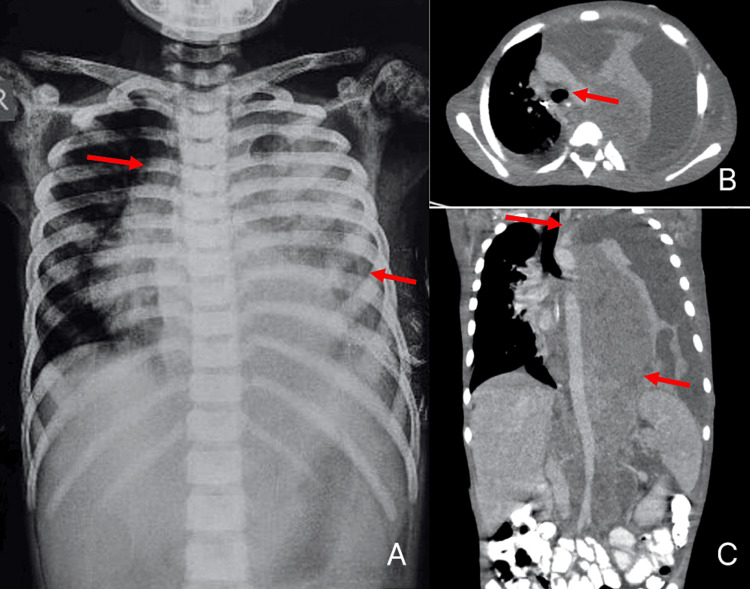
(A) Chest X-ray AP view. (B) CT thorax axial section indicating bronchial compression of less than 30%. (C) Contrast-enhanced CT thorax coronal section illustrating the extent of the mass. (A) Chest X-ray showing a mediastinal mass. (B) CT thorax axial section reveals the mass displacing the left hilum anterosuperiorly and compressing the left segmental bronchi and distal main bronchus. (C) Contrast-enhanced CT thorax coronal section displaying a large, heterogeneous soft tissue mass (28-48 HU) in the pre-vertebral, paravertebral, vascular mediastinal compartments, and retroperitoneum. The mass extends from the aortic arch to the aortic bifurcation (T4-L5), spanning both sides of the midline (left > right), measuring approximately 25 × 10 × 11 cm (CC × AP × TR). It exhibits heterogeneous post-contrast enhancement, with extensive non-enhancing necrotic areas and few coarse calcifications.

A seven-year-old boy weighing 27 kg presented to the casualty department with a cough and breathlessness persisting for 10 days. General practitioners diagnosed left-sided pleural effusions and initiated empirical anti-Koch’s treatment (AKT). After multiple pleural tap procedures, the effusion failed to resolve, leading to the insertion of an intercostal drain. The patient was referred to a tertiary care center for further evaluation. Upon admission, the patient’s oxygen saturation in room air was 94%. Pleural tapping yielded negative results for the cartridge-based nucleic acid amplification test, leading to the discontinuation of AKT. A CT thorax revealed a large, heterogeneous mass spanning the pre-vertebral and para-vertebral mediastinal compartments, extending retroperitoneally from the aortic arch to the bifurcation of the aorta (T4-L5). The mass measured 25 × 10 × 11 cm and displayed varied post-contrast enhancement with necrotic areas and coarse calcifications (Figure 1).

Predominantly on the left, the mass compressed the left hilum and bronchi, causing left lung collapse and elevating the left hemi-diaphragm. Bilateral pleural deposits and left-sided pleural effusions indicated metastatic spread. The mass encased the descending aorta along its entire length, including paired and unpaired aortic branches in the thorax and abdomen, and partially encased the inferior vena cava, displacing the renal veins and mesenteric vessels anteriorly. Enlarged lymph nodes with necrosis were present, as well as skeletal metastases in the ribs, vertebrae, left and right humeral heads, scapula, pelvic bones, and femur.

The patient was scheduled for a tru-cut biopsy of the mass. Given the mass’s proximity to major vessels and the trachea, the decision was made to keep the patient on spontaneous ventilation under sedation. After obtaining consent and confirming the patient’s nil-by-mouth status, standard American Society of Anesthesiologists (ASA)-recommended monitors were attached, and intravenous access was established. Emergency intubation preparations were made. The patient received intravenous glycopyrrolate (0.004 mg/kg) and ketamine (1 mg/kg) in the preoperative room. Spontaneous ventilation was maintained with sedation using intravenous propofol (up to 1 mg/kg) and inhalational sevoflurane (dial setting 1.0%), supplemented with intravenous paracetamol (15 mg/kg) for analgesia. The patient was positioned at a 45° head-up angle for comfort and procedure facilitation. Local anesthesia with 2% lignocaine (2 ml) was administered subcutaneously at each biopsy site. The biopsy was performed smoothly three times with a biopsy gun. The procedure was uneventful, and following full consciousness recovery, the patient was transferred to the pediatric intensive care unit (PICU) for observation until postoperative day 1.

**Figure 2:**
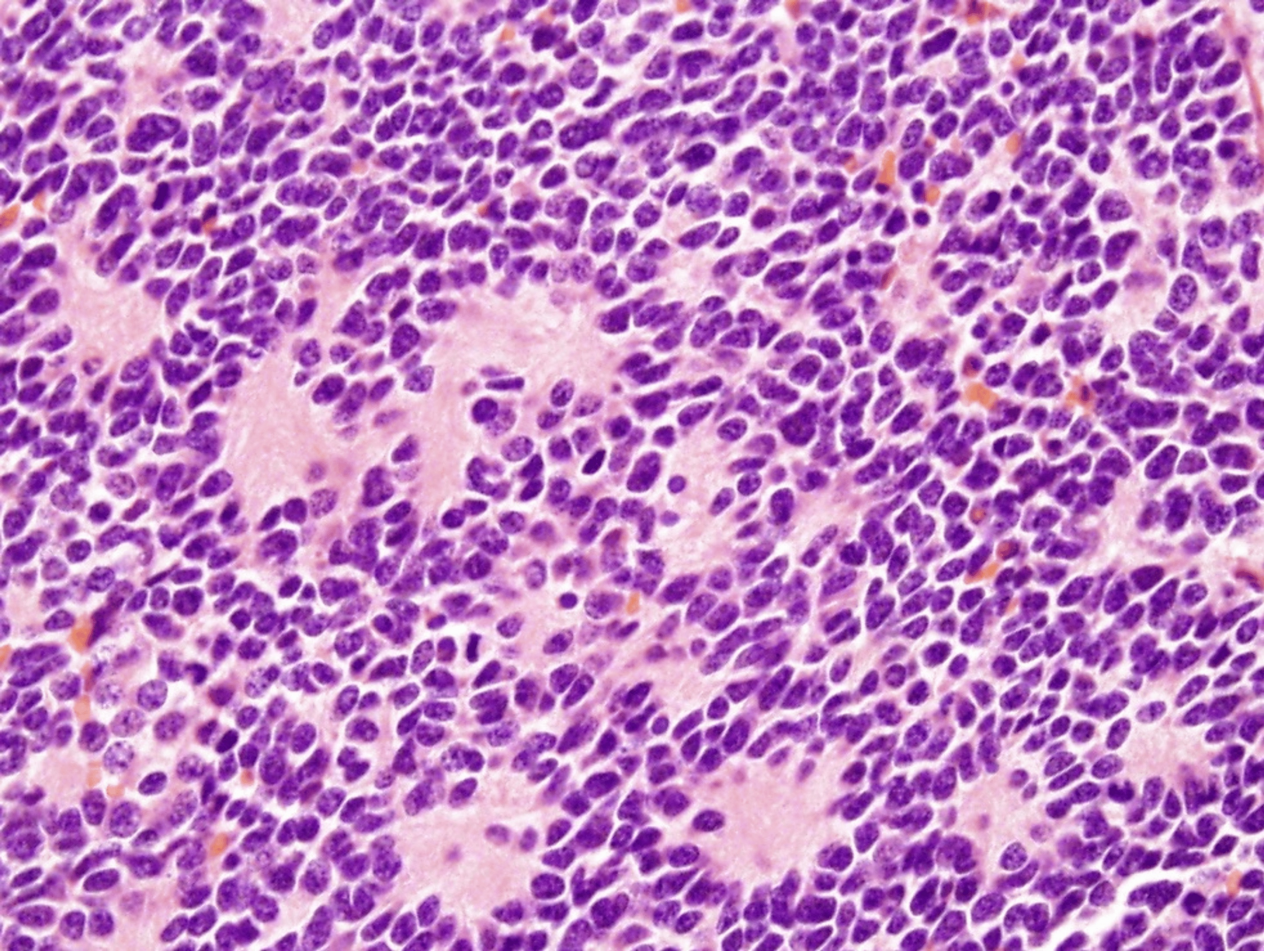
Histopathology of the mediastinal mass showing neuroblastoma with pseudorosettes. The image (H&E stain) shows a dense proliferation of small, round, blue cells with scant cytoplasm, characteristic of neuroblasts. The presence of these cells, along with Homer-Wright rosettes and neuropil, supports the diagnosis of adrenal neuroblastoma.

The histopathological examination (HPE) report of the mediastinal mass revealed neuroblastoma (Figure 2).

Case 2

**Figure 3:**
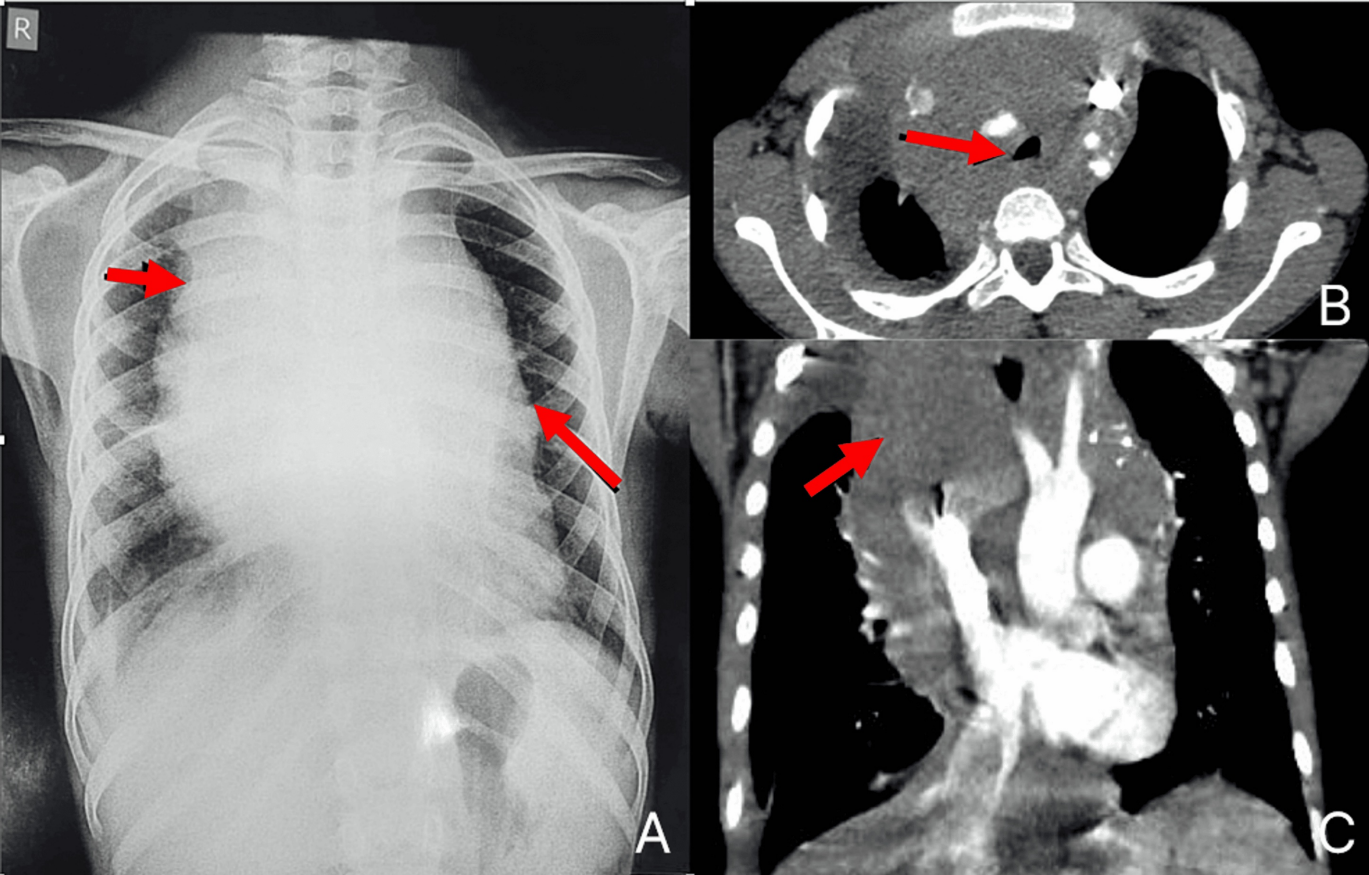
(A) Chest X-ray in PA view. (B) HRCT thorax axial section demonstrating tracheal deviation and compression. (C) HRCT contrast coronal section showing the extent of the mass. (A) Chest X-ray showing the mediastinal mass. (B) HRCT thorax axial section demonstrating tracheal compression by the mediastinal mass. (C) HRCT contrast coronal section revealing the mass, approximately 11.3 × 6 × 11.9 cm (CC × AP × TR), encasing the aorta, superior vena cava, both innominate veins, proximal bilateral subclavian arteries, left common carotid artery, brachiocephalic trunk, azygos vein, bilateral internal mammary vessels, and pulmonary arteries. The mass extends into the pre-tracheal, para-tracheal, retro-tracheal spaces, aortopulmonary window, and pre-vascular space, showing mild near-homogeneous enhancement with areas of necrosis. The superior vena cava is compressed anteroposteriorly and displaced posteriorly, while the azygos vein is displaced laterally. HRCT, high-resolution CT

A 13-year-old boy weighing 34 kg presented with retrosternal chest pain, coughing, difficulty breathing during physical activity, paroxysmal nocturnal dyspnea, fever, and acute syncope over the past six days. He also reported swelling in the neck for the past six months and had been diagnosed with tuberculosis, for which he started treatment a month ago. On examination, his pulse rate was 126 beats per minute, and his room air saturation was 98%. A CT thorax revealed mild pleural effusion in the right hemithorax and a diffuse soft tissue lesion spanning the anterior, middle, and posterior mediastinum, measuring approximately 11.3 × 6 × 11.9 cm (CC × AP × TR). These lesions compressed and displaced the aorta, great vessels, superior vena cava, and trachea (Figure 3).

Due to tracheal compression, the patient experienced difficulty breathing while lying supine. A tru-cut biopsy was performed under local anesthesia with minimal sedation, maintaining spontaneous ventilation as in Case 1. The procedure proceeded smoothly without complications, and the patient was transferred to the PICU for observation until postoperative day 1.

**Figure 4:**
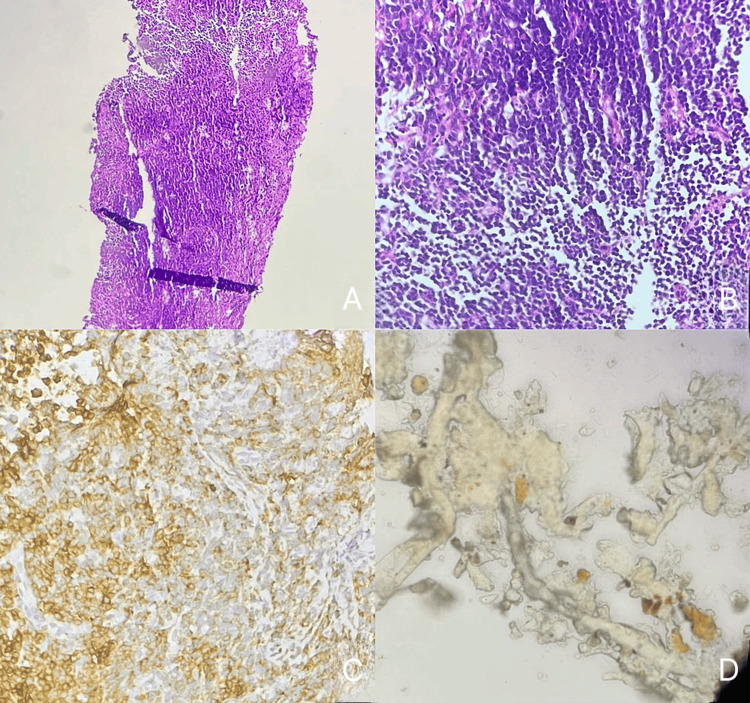
(A) T-cell lymphoblastic lymphoma at 10x magnification. (B) T-cell lymphoblastic lymphoma at 40x magnification. (C) T-cell lymphoblastic lymphoma showing CD5 marker expression. (D) Cytology of T-cell lymphoblastic lymphoma. (A and B) H&E stain reveals lymphoblasts with a high nuclear-to-cytoplasmic ratio, dispersed chromatin, and prominent nucleoli, indicating lymphoblastic lymphoma. (C) Immunohistochemistry shows positive staining for T-cell markers CD5 or TdT, confirming T-cell lineage. (D) A cytological smear displays lymphoblasts with similar features—high nuclear-to-cytoplasmic ratio, fine chromatin, and prominent nucleoli—supporting the diagnosis of lymphoblastic lymphoma.

The HPE report of the mediastinal mass revealed acute T-cell lymphoblastic lymphoma (Figure 4).

Case 3

**Figure 5:**
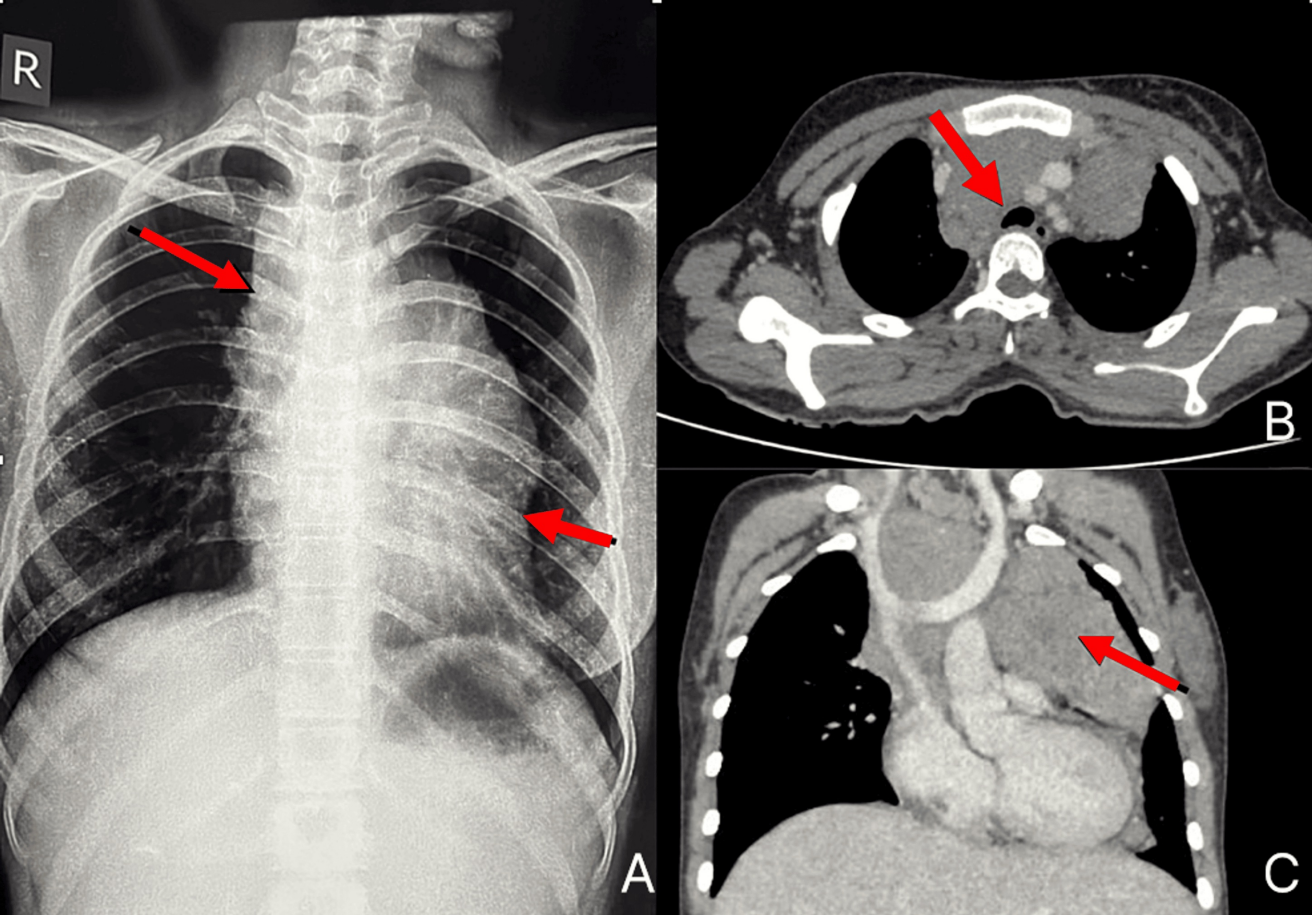
(A) Chest X-ray in PA view. (B) HRCT thorax axial section demonstrating tracheal compression and deviation. (C) HRCT contrasts the coronal section, illustrating the extent of the tumor. (A) Chest X-ray showing a mediastinal mass. (B) HRCT axial section demonstrating approximately 30% tracheal compression. (C) HRCT coronal section reveals a heterogeneously enhanced lobulated mass measuring approximately 4.7 × 6.1 × 6.8 cm (CC × AP × TR) in the prevascular region. The mass exhibits mild enhancement with a central non-enhancing area and partially abuts the arch of the aorta (~90°) while maintaining fat planes. It compresses the posterior aspect of the pulmonary trunk with the effacement of fat planes. Multiple enlarged lymph nodes with homogeneous mild contrast enhancement are present in the bilateral cardiophrenic angles, the largest measuring approximately 28 × 26 mm in the left costo-phrenic angle. A well-defined, lobulated nodule measuring 13 mm with subtle post-contrast enhancement is noted in the posterior segment of the right upper lobe. HRCT, high-resolution CT

A 14-year-old female weighing 40 kg presented with a one-month history of high-grade fever and a persistent dry cough. Despite treatment, her symptoms did not improve, leading to further evaluation. A CT scan revealed a large, heterogeneously enhancing, lobulated mass measuring 4.7 × 6.1 × 6.8 cm in the pre-vascular region. This mass partially abutted the arch of the aorta and compressed the posterior aspect of the pulmonary trunk. Multiple enlarged lymph nodes were noted in the superior mediastinum, extending into the anterior mediastinum. The mass encased the ascending aorta and brachiocephalic trunk, compressed the innominate vein, and caused the lateral displacement of the right internal jugular vein (Figure 5).

Due to the proximity of critical structures, the procedure was planned under sedation with preoperative nebulization and steroid cover. After obtaining consent and ensuring nil-by-mouth status, standard ASA-recommended monitors (ECG, BP, and SpO₂) were attached, and intravenous access was established. Preparations for emergency intubation were made, and premedications, including intravenous glycopyrrolate (0.004 mg/kg), intravenous midazolam (0.02 mg/kg), and intravenous ondansetron (4 mg), were administered. Sedation was achieved with graded doses of intravenous propofol (up to 1 ml/kg) and inhalational sevoflurane (dial at 1.0%) while maintaining spontaneous respiration. To facilitate the tru-cut biopsy by interventional radiologists, the patient was given a 45° head elevation. Local anesthesia (2% lignocaine, 3 ml) was administered subcutaneously at the biopsy site.

During the biopsy, a cough reflex triggered laryngospasm, causing a drop in saturation to 40%. Despite deepening anesthesia with intravenous propofol (2 mg/kg) and increasing sevoflurane to dial 2.0%, no air entry was achieved. Intravenous succinylcholine (1 mg/kg) was administered to relieve the laryngospasm. Ventilation was established for about 30 seconds, resulting in a momentary increase in saturation to 60% and an EtCO₂ reading of 50 mmHg. Subsequently, saturation fell to 35%, with no EtCO₂ reading. Immediate intubation with a 6 mm endotracheal tube was performed, but despite intubation and positive pressure ventilation, saturation remained unrecordable with no EtCO₂ graph, chest rise, or air entry on auscultation. Bradycardia (HR <30) ensued and was promptly treated with intravenous atropine (0.01 mg/kg). Suspecting tracheal collapse, the patient was repositioned to the left lateral position, which resolved the ventilation issues and allowed for recordable SpO₂ and EtCO₂, confirmed by auscultation. The procedure continued cautiously in the left lateral position under USG guidance. Post-procedure, the patient was extubated, received nebulization, and was monitored in the PICU overnight before being discharged to the ward the following day.

**Figure 6:**
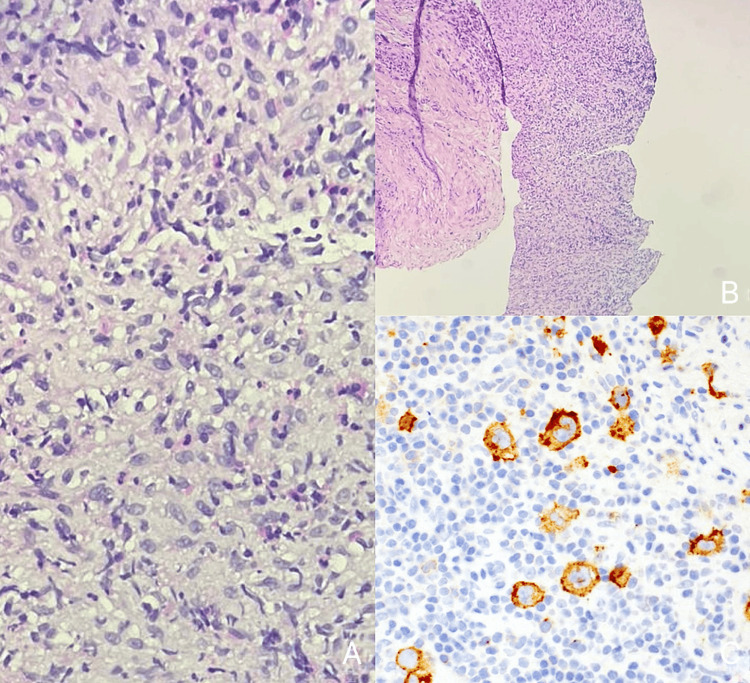
(A) Histopathology of Hodgkin lymphoma at 40X magnification. (B) Histopathology of Hodgkin lymphoma at 10X magnification. (C) Immunohistochemistry showing positive CD30 staining in Hodgkin lymphoma. (A) H&E stain reveals Reed-Sternberg cells, large abnormal lymphocytes with binucleate or multinucleate forms and prominent eosinophilic nucleoli, characterized by an “owl’s eye” appearance. These cells are indicative of Hodgkin lymphoma. (B) H&E stain shows a mixed inflammatory background with lymphocytes, eosinophils, and plasma cells. The presence of these cells alongside Reed-Sternberg cells supports the diagnosis of Hodgkin lymphoma. (C) Immunohistochemistry demonstrates positive CD30 staining (brown) in the membrane and Golgi zone of Reed-Sternberg cells, confirming their presence and the diagnosis of Hodgkin lymphoma.

The HPE report of the mediastinal mass revealed Hodgkin lymphoma (Figure 6).

## Discussion

The upper airway is composed of three segments: supraglottic, glottic, and intra-thoracic (thoracic trachea and bronchi). The mediastinum is bordered by the sternum on the anterior, with its middle section containing the heart and major blood vessels. It is limited by the thoracic inlet posteriorly, while the diaphragm forms its lower boundary. Anterior mediastinal masses can originate from the thymus, thyroid, lungs, or various other tissues and can be benign or malignant, compressing nearby structures such as the trachea, mainstem bronchi, esophagus, superior vena cava, recurrent laryngeal nerve, and heart, which are vital. This compression can lead to a range of clinical signs and symptoms, including cough, difficulty breathing (dyspnea), superior vena cava syndrome, difficulty swallowing (dysphagia), hoarseness, cardiac tamponade, and syncope or postural symptoms [1]. Additionally, patients might exhibit systemic symptoms due to the mass or the underlying pathology associated with the mass.

Anesthetic management of mediastinal mass operations can be challenging due to the risk of mediastinal mass syndrome caused by mechanical compression of mediastinal structures involving acute respiratory and hemodynamic decompensation. Currently, there are no established guidelines for managing patients undergoing mediastinal mass surgery.

During general anesthesia in patients with mediastinal masses, the most feared complications are complete airway obstruction and cardiovascular collapse [2-4]. Severe hypoxia and hypotension can occur due to compression on major airway and cardiovascular structures, respectively, such as the heart, pulmonary artery, and superior vena cava, which can occur unpredictably at any phase of anesthesia (pre-induction, induction, positioning, surgery, emergence, extubation, or even during the postoperative period in the post-anesthesia care unit) [2,3,5]. Intraoperative issues are primarily due to the mass effect on the trachea and major blood vessels. The incidence of these complications ranges from 7% to 20%, with a higher prevalence in the pediatric population [6]. Research indicates that maintaining spontaneous ventilation during the procedure can help keep the airway open [7].

Case reports and series continue to be crucial for the management of mediastinal masses, given the lack of large-scale studies. For instance, in children with significant tracheal compression, a tailored approach involving awake fiberoptic intubation or inhalation induction with the patient in a semi-sitting position is often recommended to avoid airway collapse [8].

Hack et al. found a poor correlation between tumor size, or the degree of compression of the trachea visible on CT scans, and clinical symptoms. They determined that stridor was the only symptom that reliably indicated a potential anesthetic complication. Additionally, reduction to less than 30% of the tracheal cross-sectional area from normal size reduction, or if bronchial compression to less than 70% was also present, raised respiratory complications were more frequent in patients [9].

Bittar’s mention of Gordon’s hypothesis highlights the potential danger of tracheal collapse if respiratory muscles become paralyzed during anesthesia induction. This stresses the need for thorough planning and caution to reduce such risks, including postponing surgery if signs of central airway obstruction are present. However, in our case, the procedure was crucial for establishing diagnosis and for further treatment [10,11].

Gardner and Royster’s observation of airway collapse during spontaneous breathing highlights the variability and unpredictability of airway dynamics in patients with mediastinal masses. This suggests that even patients who appear to maintain adequate ventilation spontaneously may still be at risk for airway compromise, as seen in Case 3, requiring immediate intervention [12].

Kafrouni et al.’s documentation of a patient’s inability to ventilate due to a mediastinal mass further emphasizes the importance of proactive measures such as positive pressure ventilation to manage airway obstruction effectively. However, in Case 3, despite establishing positive pressure ventilation, there was a collapse in ventilation after 30 seconds. Repositioning the patient aided in alleviating the ventilation issues [13].

Béchard et al.’s review of anesthesia cases reinforces the notion that while positive pressure ventilation may mitigate the risk of airway collapse during surgery, postoperative complications can still arise, particularly when patients return to spontaneous breathing while awake. This underscores the need for vigilant monitoring and prompt intervention to address any ventilatory complications that may arise in this patient population, which signifies postoperative vigilant monitoring for 24 hours [14].

Use of pre-medication such as antisialogogue and for sedation injection midazolam, injection ketamine, and injection propofol showed no complications in Ng et al.’s study, in which 13 out of 63 received local anesthesia or sedation for diagnostic purposes, whereas patients with more than 50% of lower-level tracheal obstruction and main bronchi suffered from sudden cardiopulmonary arrest [15].

The use of extracorporeal membrane oxygenation or cardiopulmonary bypass for patients with a mediastinal mass undergoing a tru-cut biopsy should be individualized based on the patient’s specific risks and the resources available. While these techniques can provide critical support in life-threatening situations, their use requires careful planning, and an interdisciplinary approach ensures readiness to manage emergencies.

## Conclusions

These cases highlight the importance of a thorough pre-procedural assessment for evaluating the location and impact of mediastinal masses on nearby structures. Emphasizing preparedness for airway emergencies, the approach should involve a step-wise, multidisciplinary strategy, starting with the least invasive techniques. Surgeons must have a preoperative contingency plan for children requiring general anesthesia for diagnosis. Overall, careful planning and proactive measures are crucial to minimizing risks in managing pediatric patients with mediastinal masses.

## References

[1] Wynne J, Markis JE, Grossman W (1978). Extrinsic compression of the heart by tumor masquerading as cardiac tamponade. Cathet Cardiovasc Diagn.

[2] Weng X, Jiang L, Zhou M (2023). Massive anterior mediastinal lipoma causing cardiac arrest in a middle-aged male: a case report and literature review. Future Cardiol.

[3] Stamme C, Lübbe N, Mahr KH, Dralle H, Karck M (1994). Mediastinal tumor and airway obstruction in general anesthesia. Case report and review of the literature [Article in German]. Anasthesiol Intensivmed Notfallmed Schmerzther.

[4] Sarkiss M, Jimenez CA (2023). The evolution of anesthesia management of patients with anterior mediastinal mass. Mediastinum.

[5] Asai T (2007). Emergency cardiopulmonary bypass in a patient with a mediastinal mass. Anaesthesia.

[6] King DR, Patrick LE, Ginn-Pease ME (1997). Pulmonary function is compromised in children with mediastinal lymphoma. J Pediatr Surg.

[7] Frawley G, Low J, Brown TC (1995). Anaesthesia for an anterior mediastinal mass with ketamine and midazolam infusion. Anaesth Intensive Care.

[8] Hartigan PM, Karamnov S, Gill RR (2022). Mediastinal masses, anesthetic interventions, and airway compression in adults: a prospective observational study. Anesthesiology.

[9] Hack HA, Wright NB, Wynn RF (2008). The anaesthetic management of children with anterior mediastinal masses. Anaesthesia.

[10] Bittar D (1975). Respiratory obstruction associated with induction of general anesthesia in a patient with mediastinal Hodgkin's disease. Anesth Analg.

[11] Gordon RA (1972). Anesthetic management of patients with airway problems. Int Anesthesiol Clin.

[12] Gardner JC, Royster RL (2011). Airway collapse with an anterior mediastinal mass despite spontaneous ventilation in an adult. Anesth Analg.

[13] Kafrouni H, Saroufim J, Abdel Massih M (2018). Intraoperative tracheal obstruction management among patients with anterior mediastinal masses. Case Rep Med.

[14] Béchard P, Létourneau L, Lacasse Y, Côté D, Bussières JS (2004). Perioperative cardiorespiratory complications in adults with mediastinal mass: incidence and risk factors. Anesthesiology.

[15] Ng A, Bennett J, Bromley P, Davies P, Morland B (2007). Anaesthetic outcome and predictive risk factors in children with mediastinal tumours. Pediatr Blood Cancer.

